# Using PET/CT Bone Scan Dynamic Data to Evaluate Tibia Remodeling When a Taylor Spatial Frame Is Used: Short and Longer Term Differences

**DOI:** 10.1155/2015/574705

**Published:** 2015-09-07

**Authors:** Henrik Lundblad, Gerald Q. Maguire, Charlotte Karlsson-Thur, Cathrine Jonsson, Marilyn E. Noz, Michael P. Zeleznik, Hans Jacobsson, Lars Weidenhielm

**Affiliations:** ^1^Department of Molecular Medicine and Surgery, Karolinska Institutet, 17176 Stockholm, Sweden; ^2^School of Information and Communication Technology, KTH Royal Institute of Technology, 16440 Stockholm, Sweden; ^3^Department of Hospital Physics, Karolinska University Hospital Solna, 17176 Stockholm, Sweden; ^4^Department of Radiology, New York University, New York, NY 10016, USA; ^5^School of Computing, College of Engineering, University of Utah, Salt Lake City, UT 84109, USA

## Abstract

Eighteen consecutive patients, treated with a Taylor Spatial Frame for complex tibia conditions, gave their informed consent to undergo Na^18^F^−^ PET/CT bone scans. We present a Patlak-like analysis utilizing an approximated blood time-activity curve eliminating the need for blood aliquots. Additionally, standardized uptake values (SUV) derived from dynamic acquisitions were compared to this Patlak-like approach. Spherical volumes of interest (VOIs) were drawn to include broken bone, other (normal) bone, and muscle. The SUV_*m*_(*t*) (*m* = max, mean) and a series of slopes were computed as (SUV_*m*_(*t*_*i*_) − SUV_*m*_(*t*_*j*_))/(*t*_*i*_ − *t*_*j*_), for pairs of time values *t*_*i*_ and *t*_*j*_. A Patlak-like analysis was performed for the same time values by computing ((VOI_*p*_(*t*_*i*_)/VOI_*e*_(*t*_*i*_))−(VOI_*p*_(*t*_*j*_)/VOI_*e*_(*t*_*j*_)))/(*t*_*i*_ − *t*_*j*_), where *p* = broken bone, other bone, and muscle and *e* = expected activity in a VOI. Paired comparisons between Patlak-like and SUV_*m*_ slopes showed good agreement by both linear regression and correlation coefficient analysis (*r* = 84%, *r*_*s*_ = 78%-SUV_max_, *r* = 92%, and *r*_*s*_ = 91%-SUV_mean_), suggesting static scans could substitute for dynamic studies. Patlak-like slope differences of 0.1 min^−1^ or greater between examinations and SUV_max_ differences of ~5 usually indicated good remodeling progress, while negative Patlak-like slope differences of −0.06 min^−1^ usually indicated poor remodeling progress in this cohort.

## 1. Introduction

The Taylor Spatial Frame (TSF) [1], an Ilizarov-derived circular frame [2], is used to treat fractures or correct skeletal deformity. The patient postoperatively applies a sequence of adjustments to the fixator, according to the orthopaedic surgeon's prescription to achieve desired alignment and/or lengthening. Therapy takes many months and the patient returns periodically for a computed tomography (CT) or planar X-ray study of the limb. This information allows the orthopaedic surgeon to modify the prescription, to decide upon a new surgical procedure, or if the bone is stable enough to remove the TSF.

It has been well established in the literature since the 1950s that bone rapidly takes up ^18^F^−^ (fluoride) and, in broken bone, this uptake is increased [3–6]. Previously [7] our group showed that ^18^F^−^ PET/CT might be valuable to study a patient's bone remodeling using standardized uptake values (SUVs) computed for a volume of interest (VOI) over the crural fracture/osteotomy and a portion of nonaffected tibia at 30, 45, and 60 minutes after ^18^F^−^ injection. This allowed the orthopaedic surgeon to follow the course of therapy, especially* early* in the treatment, whether or not there was a need for a new surgical procedure, or* late* in the treatment whether the TSF could be removed.

The present study utilized dynamic (list mode) data to assess the time dependence of ^18^F^−^ uptake in a VOI over the crural fracture/osteotomy and in reference tissues (normal bone and muscle). Based on prior work on irreversible tracers [8, 9] and the rapid uptake of ^18^F^−^ by remodeling bone [6, 10, 11] and approximating the expected activity in a VOI due to diffusion, a Patlak-like analysis was performed. As the goal was to understand patient specific uptake without requiring blood sampling, the aim is similar to Sayre et al.'s Patlak-P [12]. Unlike Blake et al. [13] who compare different therapies across patients, this study focuses on improving the treatment of specific patients. We show that a Patlak-like analysis (without actual blood aliquots) is sufficient to determine bone remodeling; SUV_mean_ and SUV_max_ data from static scans performed at specific times can substitute for a dynamic scan; and the examination time can be shortened if scanner time is limited.

## 2. Methods and Materials

### 2.1. Patients

Eighteen consecutive patients (4 females) who had a TSF applied to the tibia gave informed consent to participate in this study (Regional Ethics Committee Dnr. 2012/1049-31/1). The mean patient age was 42 (range 18–68) years. The patients were examined at approximately 35 days (range 39–61, mean 46) after TSF surgery and again at approximately 90 days (range 82–128; mean 104). The reasons for the delayed studies were that in some cases the patient was not available at the exact six-week or three-month time frame or that there were technical difficulties with the cylotron or the PET/CT scanner.  Table 1 describes each patient, along with days since the TSF was attached until the first and second PET/CT. There were only 39 (of 44 possible) lists available. Patients 8 and 9 who failed to heal were reexamined twice after revision surgery without removal of the original TSF. Thus Patient 8 had 2 extra lists. However, three out of four lists for Patient 9 and one out of two lists for Patient 6 were not available due to technical acquisition error. Patient 1, who was examined shortly before TSF removal, had another TSF applied and was reexamined. The original proximal and distal crural fractures healed; however, this patient suffered a further break between the original two. This fracture was not treated with a TSF, but for clinical reasons the patient returned for additional PET scans allowing evaluation of the previous “healed” fractures [14] resulting in 3 extra lists. Patient 15 had 1 extra list. Patient 2 was only examined once. Patient 5, who suffered from genu varum, had both tibiae treated allowing the list to be examined for each leg. Thus we were able to perform 41 list analyses.

### 2.2. [^18^F^−^ Fluoride] PET/CT Bone Scan

All patients were examined using a clinical PET/CT scanner (Biograph 64 TruePoint TrueV, Siemens Medical Solutions, Erlangen, Germany). One bed position (20 cm), centered at the location of the crural fracture/osteotomy, was used. After hydration with 7 deciliters of water 30 minutes prior to the examination, the patient was positioned on the scanning couch as previously described [15]. A noncontrast, diagnostic CT was performed as described in Table 2 [15]. A list mode PET acquisition was started simultaneously with the intravenous Na^18^F^−^ injection (2 MBq/kg body weight) and continued for 45 minutes. Volumes were reconstructed as described in Table 2 for intervals from injection time to 1, 2, 3, 4, 5, 8, 11, 14, 17, 20, 25, 30, 35, and 45 minutes after injection. These times were chosen as described in Discussion.

### 2.3. Dynamic Scan Analysis

Our previously described and validated [16–18] 3D image processing software tool was used for the SUV and Patlak-like analysis. For each available list mode volume, a 3D spherical VOI (25 mm radius, 65.45 mL volume) was created around the crural fracture/osteotomy on the 45-minute volume (VOI_broken  bone_). The location of the crural fracture/osteotomy was confirmed by superimposition of the registered CT data on the PET data. Additional VOIs of the same radius were created usually on the contralateral tibia designated VOI_other  bone_ and in muscle designated VOI_muscle_. These VOIs were used to generate the data required for the Patlak-like analysis and the SUV_max_ and SUV_mean_.

For the Patlak-like analysis an approximation was made of the activity that would be expected at a time *t* in a VOI due to diffusion of the injected radionuclide, VOI_*e*_(*t*). To obtain these data it was assumed that the radioactivity being transported by the blood and transferred to the interstitial fluid decreased during the whole acquisition at the same rate as the physical decay of ^18^F^−^. The fact that this is a plausible conjecture can be seen from the decay curves presented in the early work of Weber [19, 20] as well as in the careful analysis by Creutzig [21]. Further at acquisition start, the radioactivity concentration in the blood was calculated by dividing the injected activity (MBq) by an assumed 5000 mL blood volume. VOI_*e*_(*t*), in units of Bq/mL, is the ^18^F^−^ decay corrected activity within a VOI sized volume of blood. Six time intervals (ending, starting), (35, 4), (35, 5), (35, 8), (35, 11), (45, 8), and (45, 11) minutes, were used for this analysis because they best illustrated the rate of uptake by the broken bone. The Patlak-like slope for each time interval, (*t*_*j*_, *t*_*i*_) minutes, was calculated as ((VOI_*p*_(*t*_*i*_)/VOI_*e*_(*t*_*i*_))−(VOI_*p*_(*t*_*j*_)/VOI_*e*_(*t*_*j*_)))/(*t*_*i*_ − *t*_*j*_), where *p* = broken bone, other bone; muscle and VOI_*p*_(*t*) = (∫_0_^*t*^rescale  slope × voxel  values_*p*_ *dt*)/volume. To check this Patlak-like data for consistency, these slopes were assessed by linear regression for the six time intervals and the slope and coefficient of determination (*R* ^2^) values were recorded for each.

Additionally, a series of slopes were computed as (SUV_*m*_(*t*_*i*_) − SUV_*m*_(*t*_*j*_))/(*t*_*i*_ − *t*_*j*_), where *m* = max and mean, for the same pairs of selected time values *t*_*i*_ and *t*_*j*_ (minutes after injection) as above. Each SUV_*m*_(*t*_*i*_) is computed based upon the SUV_*m*_ for the interval from 0 to *t*. Histograms, box, density, and quantile-quantile plots were used to check that the slope data were normally distributed (Gaussian distribution). Although the data were nearly normal, the nonparametric Spearmen correlation coefficient (*r*_*s*_) and the Pearson correlation coefficient (*r*) were used to evaluate the correlation of the SUV_*m*_ slope with the Patlak-like slope. SUV_*m*_ slopes were plotted against the Patlak-like slopes for each time interval, linear regression analysis was performed, and a regression line was added to the plot. The open source statistical package R version 3.0.2 was used for all statistical calculations and plots [22].

## 3. Results

For all patients at least one dynamic list was available. It was feasible to perform the analysis for all available list data.

### 3.1. Patlak-Like Results

The average linear regression *R* ^2^ values for the broken bone were 0.98 for the first two time intervals and 0.99 for the last four intervals, showing that each Patlak-like slope was consistently linear. The average *R* ^2^ values for other bone ranged from 0.94 to 0.96 and for muscle ranged from 0.71 to 0.86.

The average values of the Patlak-like slopes for the broken bone for the six time intervals ranged from 0.25 min^−1^ to 0.21 min^−1^, while the average values for other bone ranged from 0.020 min^−1^ to 0.015 min^−1^, and the slope for muscle ranged from 0.011 min^−1^ to 0.006 min^−1^. All of these values generally decrease as time increased.

### 3.2. SUV Results versus Patlak-Like Results

**Figure 1:**
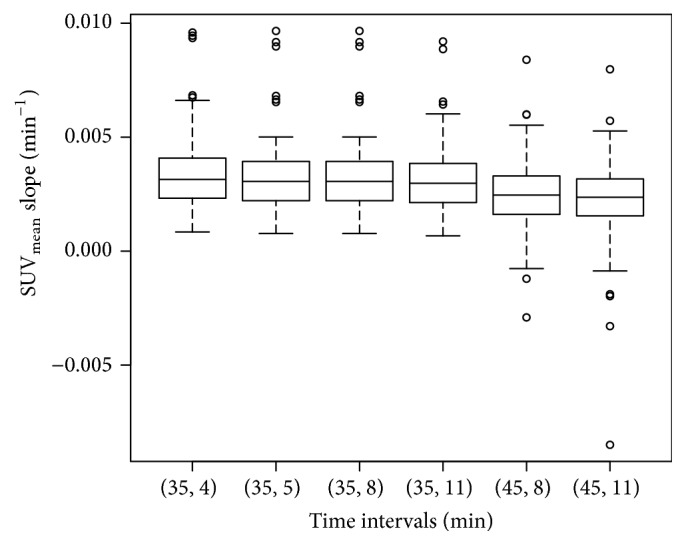
The SUV_mean_ data is shown to be nearly normal, especially for the intervals 11 to 35 and 8 to 45 minutes.

**Figure 2:**
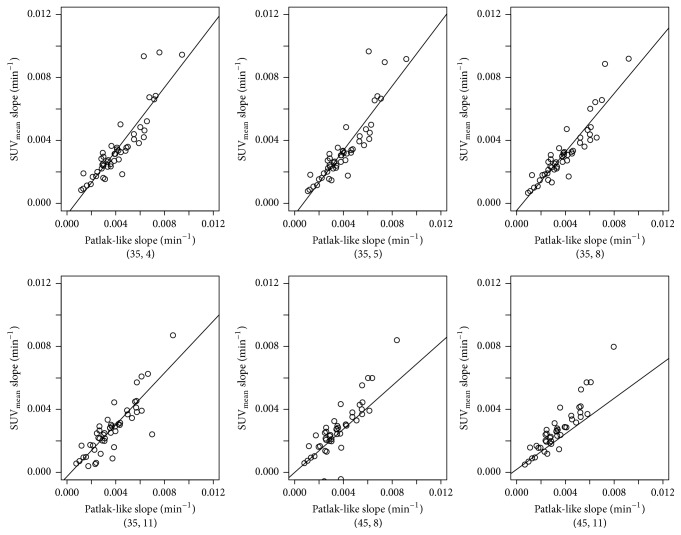
Plot of Patlak-like slope versus SUV_mean_ for different time intervals with regression line superimposed, showing a linear relation between the two values.

The slope for the SUV_mean_ and SUV_max_, respectively, calculated over the same time intervals was consistently nearly normally distributed as shown in Figure 1 for the SUV_mean_. As shown in Figure 2 a plot of the SUV_mean_ slope data against the Patlak-like slope data demonstrates that they are linearly related with an *R* ^2^ value ranging from 0.85 to 0.86, an average Pearson correlation coefficient *r* of 0.92 (range 0.92–0.93), and an average Spearman correlation coefficient *r*_*s*_ of 0.91 (range 0.90–0.91). For the SUV_max_ slope data, the *R* ^2^ value ranged from 0.78 to 0.84, average *r* = 0.84 (range 0.83–0.85), and *r*_*s*_ = 0.78 (range 0.73–0.81).  Table 3 gives a summary of both Patlak-like and SUV_max_ and SUV_mean_ results for each patient.

### 3.3. Example Demonstrating the Effect of Choice of VOI_*e*_(*t*) on the Patlak-Like Approach

**Figure 3:**
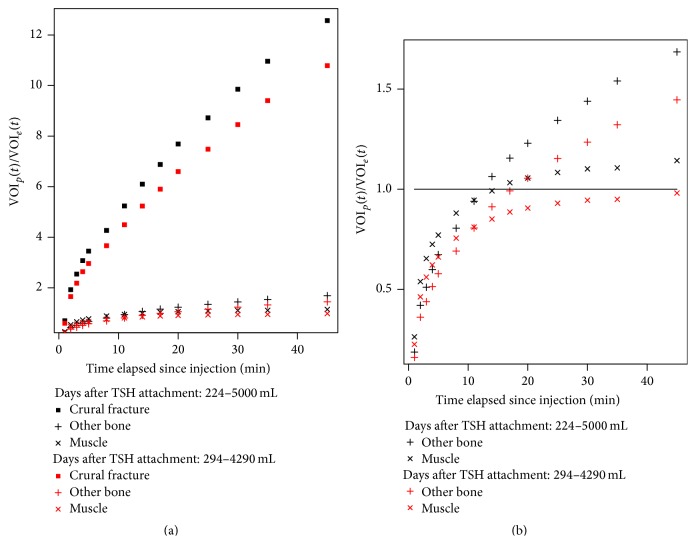
(a) VOI_*p*_(*t*_*i*_)/VOI_*e*_(*t*_*i*_) for *p* = broken bone, other bone, and muscle as a function of time since the injection for Patient 9 for two different estimates of total blood volume. (b) shows the same ratio for only other bone and muscle. The line shows that VOI_muscle_(*t*_*i*_)/VOI_*e*_(*t*_*i*_) at both blood volumes is close to one.

Figure 3(a) shows Patient 9's ratios VOI_*p*_(*t*)/VOI_*e*_(*t*) for *p* = broken bone, other bone, and muscle when using VOI_*e*_(*t*) based upon a blood volume of 5000 mL and for a 4290 mL blood volume. The later blood volume was estimated based upon the weight of the patient (66 kg) multiplied by the estimated blood volume per kg of an adult female (65 mL/kg, computed from Table 3–5 of [23]). As each patient's height was unknown, Nadler's formula could not be used. In both cases VOI_*e*_(*t*) is calculated as if the VOI was filled with blood.

Removing the broken bone ratios from the graph allows the smaller ratios of other bone and muscle to be seen more easily; see Figure 3(b). Note that at 45 minutes the ratio for muscle is slightly above one for an estimated blood volume of 5000 mL, while that ratio for an estimated blood volume of 4290 mL is slightly below one.

### 3.4. Specific Examples

**Figure 4:**
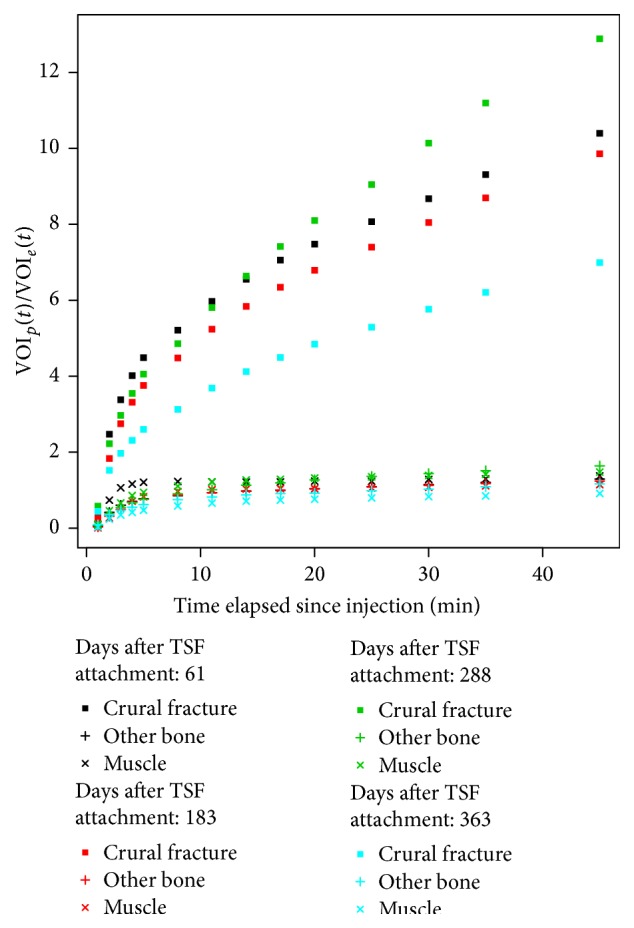
Patlak-like curves for Patient 8 before (days after TSF attachment 61 and 183) and after revision surgery (days after TSF attachment 288 and 363).

Patient 8 sustained a gunshot wound to the distal third of his left tibia and fibula. The fracture was fixed with an intramedullary nail and the patient presented five months later to the reconstruction section with an infected pseudarthrosis and a foreign body remaining in the soft tissue. He was revised with intramedullary reaming, extraction of the foreign body, application of Gentamycin, and fixation with a TSF. Sixty-one days postoperatively the 45-minute scan had a SUV_max_ of 31.1 and at 183 days the SUV_max_ was 36.0. He was followed with plain film X-rays and was fully weight bearing and painless. However, a CT scan showed a hypertrophic nonunion. After 244 days from the original operation, he was revised with an osteotomy for lengthening of the tibia proximally, bone grafts, and compression/stabilization of the nonunion, without removal of the original TSF. His subsequent ^18^F^−^ PET/CT scan 288 days after TSF attachment had a SUV_max_ of 35.9 and at 363 days the SUV_max_ had fallen to 25.0. He had the TSF removed at 413 days and commenced dancing lessons.  Figure 4 shows the Patlak-like analysis for this patient.

**Figure 5:**
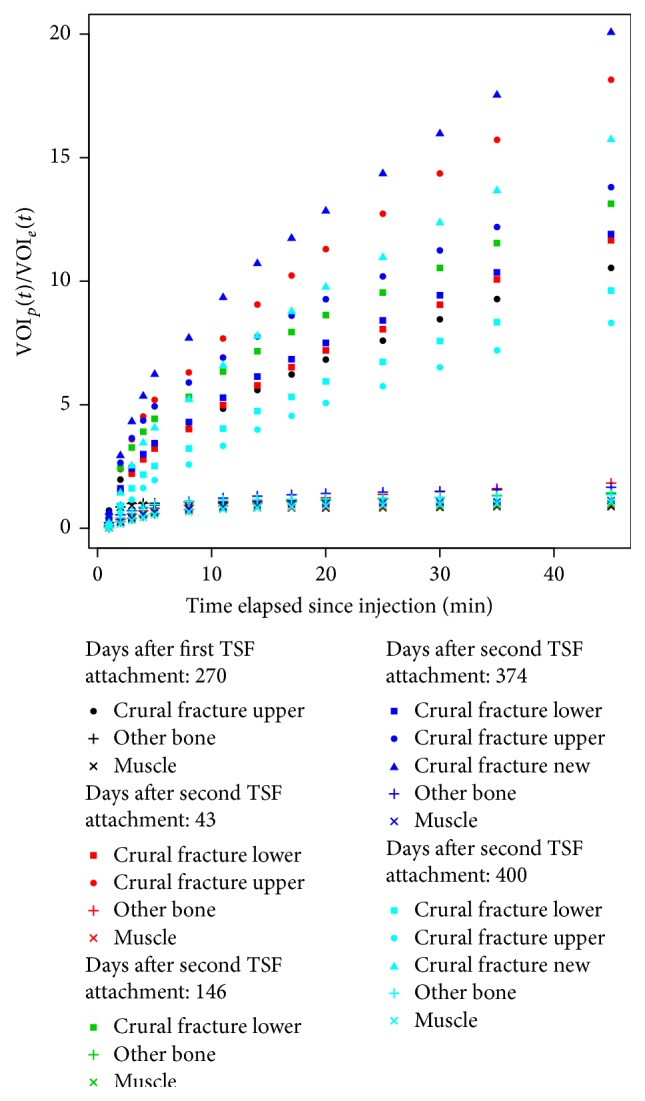
Patlak-like curves for Patient 1 at five different points in time, both before and after removal of the TSF.

Patient 1 was followed before and after removal of the second TSF. He had refractures in an open segmental tibial fracture and was treated first with a two-level TSF without revision. The first ^18^F^−^ study was performed to aid the decision to extract the frame. In the 45-minute scan, a SUV_max_ of 38.8 in the proximal tibia indicated an ongoing high bone turnover indicating ongoing healing of the bone, confirmed by the morphological distribution of uptake. The TSF was removed after 323 days at the patient's request; a cast was applied, but subsequently the patient had a refracture in the intermediate fragment and a varus dislocation that required further treatment with a TSF. The refracture was “activated” by drilling and a proximal osteotomy was done for gradual correction of the varus deformity and a slight lengthening. At 43 days from the attachment of the second TSF, the 45-minute scan showed a SUV_max_ of 54.0 in the proximal tibia and 38.4 distally and at 146 days the distal tibia had increased to a SUV_max_ of 48.0. This second TSF was removed after 168 days. The patient then again developed a fracture in the intermediate segment that this time was treated with an intramedullary nail. For clinical reasons, he had two more PET/CT examinations at 374 and 400 days from attachment of the second TSF. The 45-minute acquisition showed the original proximal tibia had a SUV_max_ of 42.2 and 18.2 at 374 and 400 days, respectively, and the original distal tibia of 47.2 and 42.7, respectively. Patlak-like analysis of this patient is shown in Figure 5. The fracture in the intermediate segment went on to healing and the patient is now walking without pain although sometimes using a crutch.

## 4. Discussion

This work shows that it is possible to take several static time points and, using SUV analysis, obtain a rate of increase of bone uptake which is comparable to that of a complete dynamic scan. As can be seen in Figure 2, the nearly linear regression line and the high correlation coefficient indicate that there is indeed an acceptable correlation between the Patlak-like and the SUV analysis. Thus, it is possible to substitute SUV analysis derived from a few static scans for the complete dynamic scan when scanner time is limited. As can be observed from Table 3, the SUV_mean_ value, the Patlak-like slope value, and the SUV_mean_ slope values are consistent: if one value increases between examinations, the other two values do as well and* vice versa*. Thus in this study SUV_max_ and SUV_mean_, respectively, as well as Patlak-like slope and the SUV_mean_ slope values were examined for short term differences between static volumes reconstructed from injection time to 4, 5, 8, and 11 minutes after injection and 35 and 45 minutes after injection on a specific date and longer term differences between serial PET/CT examinations (*n* = 15). For example, in Figure 4, showing the results for Patient 8, the difference in his rate of uptake between his first two scans (black and red curves) was negligible. This suggested that his healing was not progressing well. However, after revision, his rate of uptake between scans (green and cyan) dramatically increased, indicating that bone remodeling was occurring. Since the bone actually healed, this is evidence that this method could be useful. The first two scans were available 183 days after the operation, but the revision was not done until 244 days. In hindsight, these results should have led to an earlier revision and the patient's total treatment time (413 days) shortened.

As expected, the average values of the Patlak-like slopes for the broken bone and other bone decreased for the six time frames consistent with the uptake* rate* of ^18^F^−^ decreasing as the bone becomes saturated; while Patlak-like slopes for the muscle decreased due to clearance of ^18^F^−^ from the blood. This has been discussed in the early work of Weber et al. [19, 20] as well as the careful studies by Creitzig in Germany [21, 24, 25] which all show that ^18^F^−^ is rapidly cleared from interstitual blood pool, as we assume in this study.

Cook et al. noted that a noninvasive, substitute method for replacing the arterial input function is very desirable [26]. The estimation of VOI_*e*_(*t*) by assuming that the activity in a VOI is simply the injected activity diluted by a fixed blood volume acting as a surrogate for collecting aliquots is consistent with the methods contrasted in Cook's study. As shown in Figure 3, for this single patient, the change in VOI_*p*_(*t*)/VOI_*e*_(*t*) introduced due to the actual blood volume not being 5000 mL is negligible as VOI_muscle_(*t*)/VOI_*e*_(*t*) was nearly 1 at 45 minutes for either blood volume. This is a simplification of the diffusion of the radionuclide that was injected into the blood into each of the VOIs and assumes equal diffusion of the radionuclide in different VOIs (all of the same volume) but enables us to compute a dimensionless ratio that is insensitive to the injected activity. This may not be true for all patients or even for a given patient at different points in time (Piert et al. describe differences in the diffusion due to blood flow [27]) and requires further investigation. Note that the computation of  VOI_*p*_(*t*) is similar to the computation of SUV_mean_(*t*) in that both results reflect averaging. Further, this study shows that an analog to a blood time activity curve can be obtained without the necessity of having drawn blood aliquots. Eliminating the aliquots makes it much easier for the staff, physicians, and patients yet yields acceptable results.

Figure 5, which shows the progress of healing for Patient 1, indicates its usefulness. Both the upper and lower refracture/osteotomy after treatment with the second TSF seem to be individually remodeling at a steady, but distinct pace, even after TSF removal. The use of ^18^F^−^ in orthopeadic investigations has been reviewed in Adesanya et al. [28] and has been addressed also in Lévy and Fenollar [29]. Although this patient group was inhomogeneous, this treatment is reserved only for difficult cases, where more conventional treatments cannot be successfully used or having been used have failed to have the patient heal properly.

The time intervals ranging from 8 to 45 minutes were initially chosen because the literature suggested that the injected activity would be well distributed in the blood within 10 minutes [30]. The Society of Nuclear Medicine's “Guideline for Sodium ^18^F-Fluoride PET/CT Bone Scan” [31] suggests that axial skeleton images can be acquired as soon as 30–45 minutes after injection and static images of 3 minutes per bed position can be acquired after 45 to 60 minutes based on traditional bone studies focused on obtaining clinically useful images for a variety of purposes (see, e.g., [32]). However, according to Kurdziel et al. the optimal uptake interval remains to be defined [33]. This study, coupled with [7], is consistent with the view that 35–60 minutes are sufficient for obtaining a clinically useful study.

Numerous studies of  ^18^F^−^ bone uptake following fractures have been done (primarily with rats and dogs) [34]. For example, Dworkin et al. showed an order of magnitude difference in the uptake between the wounded leg and unwounded leg of a dog [6]. Further, ^18^F^−^ uptake is governed by regional blood flow and osteoblastic activity. Czernin et al. [11] described the molecular mechanisms of  ^18^F^−^ deposition in bone noting that blood flow is the rate limiting step of uptake and showed, citing [35], that almost all of the ^18^F^−^ is retained from a single pass of the blood; only 10% of ^18^F^−^ is in the blood an hour after injection, as ^18^F^−^ is cleared rapidly from the blood (by both bones and kidneys). The uptake and retention of ^18^F^−^ is a function of the “exposed” bone surface (suggesting that this surface interacts with the extracellular fluid, hence the site of the incorporation into the bone). Raijmakers et al. have recently compared a number of different clinically useful methods of measuring bone metabolism and bone blood flow with full kinetic analysis and shown that both Patlak and SUV methods could be used for assessing fluoride kinetics in humans [36]. Similar results were obtained earlier by Frost et al. [37]. We observed nearly a factor of 5 difference between the uptake rates of the* healing* broken bone versus other bone and muscle. This provides critical information to the orthopaedic surgeon who needs to know if the broken bone is* not* healing, if so some remedial action is needed.

**Figure 6:**
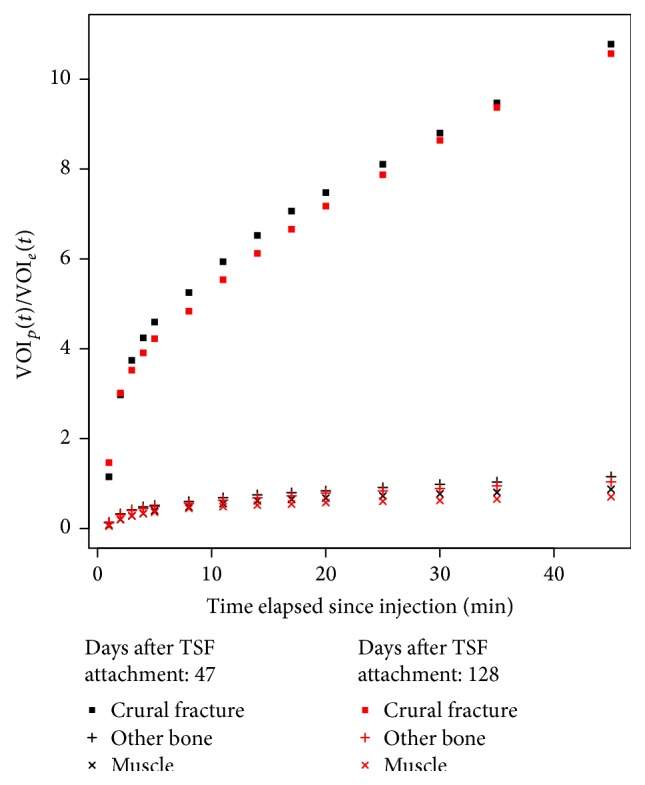
Patlak-like analysis of Patient 7 who had recurrent osteomyelitis in fracture region shows a rapid uptake of  ^18^F^−^ initially, with the onset of the reduced rate of uptake very early in the 45-minute interval.

There has been some recent work on early dynamic ^18^F^−^ bone scanning [38]. Freesmeyer et al. studied the uptake of ^18^F^−^ in the case of chronic osteomyelitis very soon after the injection and found increased uptake in frames 31 to 45 seconds after injection [38]. Similarly, in this study, most patients who did not have osteomyelitis were observed to have increased uptake rates in the VOIs over the affected bone in the first several minutes and a reduction in this rate later in the mid-phase (25 to 35 minutes) of the dynamic scan. An example of this change in rates of uptake can be seen in Figures 3, 4, and 5. However, for Patient 7 who had a chronic osteomyelitis the early Patlak-like analysis showed a rapid uptake with a decreased rate beginning at 5–8 minutes as shown in Figure 6.

Referring to the kinetic parameters *k*_2_, *k*_3_, and *k*_4_, Wong and Piert state “The magnitude of *k*_4_ (the non-reversible, hence consistent with Patlak analysis, parameter) is typically small in comparison to *k*_2_ and *k*_3_, indicating little dissociation of fluoride from the bone matrix.” [39]. There was no example in this study of any significant decrease in VOI_broken bone_(*t*) or VOI_other bone_(*t*) with increasing *t*, suggesting that there is no dissociation of the fluoride from the bone as was also reported by Blake et al. [40]. The data presented here, as exemplified in both the Patlak-like curves and the time frame SUV_mean_, are consistent with no dissociation of the fluoride from the bone (*k*_4_ = 0); therefore the Patlak and Blasberg [8, 9] graphical method can be used to estimate bone remodeling.

Limitations of this study are that the arterial input function was not obtained during the PET acquisitions and a regular Patlak analysis could therefore not be performed for purpose of comparison with the present Patlak-like method. The estimated time-activity-curve for blood was calculated based on the injected activity, an assumed 5000 mL blood volume, and a decrease in activity similar to that of the physical decay of ^18^F^−^.

## 5. Conclusions

The correlation between the SUV_mean_ versus Patlak-like slope analysis for intervals over the first 35 minutes correlated well, suggesting that 35 minutes might be a sufficient study time rather than 45 or 60 minutes, if longer scan times are not feasible. The dynamic analysis, as performed in the present study, was* not* superior to simple SUV measurements, suggesting that full dynamic analysis may be* unnecessary* as sufficient clinical information can be obtained from SUV analysis alone. The longer term differences of this larger cohort of patents confirms the results of [7] with regard to assessing the patient's bone remodeling. We are currently investigating what additional information might be obtained from analysis and visualization of the dynamic 3D data from each acquisition.

## Figures and Tables

**Table 1:** Patient description (N/A means not applicable).

Patient	Age	Sex	Days first PET/CT	Days second PET/CT	Reason	Resolution	Days TSF applied
P1	64	M	274	N/A	Refracture in segmental tibial left	TSF extraction	328
P1	64		43	146	New TSF as fractures not healing		168

P1	64	M	374	400	New fracture between former two	Former two fractures remodeling	N/A

P2	36	M	135	N/A	Pseudarthrosis right lower leg	TSF extraction healed	211
P3	52	M	40	84	Fracture healing in left leg		167
P4	44	M	50	122	Pseudarthrosis right lower leg		161
P5	35	M	43	85	Genu varum, pseudoachondroplasia		182
P6	17	F	52	94	Reduction malformation right leg		345

P7	31	M	48	129	Osteomyelitis right lower leg fracture	Leg amputated, continued infection	226

P8	28	M	60	184	Pseudarthrosis left lower leg	Patient did not heal, new operation	N/A

P8	28	M	288	363	Reoperated no new TSF was applied	TSF extraction healed, dancing	413

P9	45	F	50	91	Nonunion/pseudarthrosis distal tibia/pilon fracture right distal tibia	CT, nonunion, plane film X-ray *not* seen. Low 50-day uptake should have prompted revision	N/A

P9	45	F	224	294	Reoperated no new TSF was applied	TSF extraction healed	355
P10	33	M	42	90	Fracture varus deformity + lengthening		106
P11	68	F	43	87	Wound autologous bone grafting		156
P12	35	M	48	104	Severe bow deformities of tibiae		151
P13	30	M	44	89	Varus deformity and lengthening		100
P14	21	F	48	94	Genu valgum, valgus deformity		115

P15	52	M	52	93	Pseudoarthrosis, osteotomy	Patient not remodeling as expected	N/A

P15	52	M	148	N/A	Reoperated no new TSF was applied	Ongoing TSF with ultrasound of bone	N/A

P16	40	M	145	184	Proximal tibia fracture, varus deformity, original scan delayed	TSF extraction healed, returned for a second scan 35 after removal	149

P17	70	M	48	82	Comminuted distal tibial fracture	TSF extraction healed	147
P18	29	M	44	83			199

**Table 2:** PET and CT reconstruction parameters.

Modality			Resolution			Pixel size (mm)		
	Parameters	Reconstruction	*X*	*Y*	*Z*	*X*	*Y*	*Z*
PET	Dynamic list mode	OSEM2D 4 iterations 8 subsets Gaussian filter 5 mm	168	168	74	4.07	4.07	3.00

CT	120/140 kVp, 50/60 mAs 0.5/1.0 second per revolution 1.0 pitch	Attenuation correction	512	512	74	1.37	1.37	3.00

**Table 3:** Summary of findings for all patients. Lo indicates distal tibia, Up indicates proximal tibia, L means left leg, and R means right leg. All SUV values at 45 minutes.

Patient	Days after TSF surgery	Operated leg SUV_max⁡_	Operated leg SUV_mean_	Patlak-like slope for operated leg at (45, 8) minutes^−1^	SUV_mean_ slope at (45, 8) minutes^−1^	Nonoperated leg SUV_max⁡_
P1	270	38.82	8.54	0.176	0.0024	2.63
P1-Lo	43	38.35	9.38	0.18	0.028	3.35
	146	48.18	11.00	0.21	0.0029	2.04
	374	47.24	9.50	0.21	0.0027	3.28
	400	42.74	7.71	0.15	0.0023	2.80
P1-Up	43	54.00	14.60	0.32	0.0043	3.35
	374	42.37	11.01	0.33	0.0028	3.28
	400	21.23	6.66	0.28	0.0021	2.80

P2	133	26.23	6.65	0.13	0.0017	2.83

P3	39	71.13	26.56	0.40	0.0072	3.33
	83	56.62	19.98	0.33	0.0055	2.60

P4	49	58.38	21.11	0.36	0.0060	2.42
	119	42.80	15.87	0.23	0.0043	3.22

P5-R	42	29.46	5.72	0.15	0.0013	4.37
	83	48.58	6.47	0.18	0.0016	3.25
P5-L	42	35.04	5.84	0.16	0.0014	4.61
	83	36.91	7.64	0.23	0.0020	2.53

P6	92	42.04	8.81	0.15	0.0022	3.15

P7	47	29.12	10.88	0.15	0.0025	3.20
	128	27.27	11.59	0.15	0.0028	2.91

P8	61	31.05	7.26	0.14	0.0016	2.00
	183	36.01	7.37	0.15	0.0018	2.20
	288	35.90	9.64	0.22	0.0027	1.50
	363	24.93	5.02	0.10	0.0013	3.33

P9	294	22.50	8.27	0.22	0.0025	2.97

P10	42	80.03	22.40	0.38	0.0060	2.94
	90	81.05	31.32	0.50	0.0084	1.54

P11	43	55.33	12.88	0.33	0.0040	2.08
	87	29.35	7.81	0.21	0.0024	1.29

P12	48	51.91	13.07	0.31	0.0033	3.94
	104	42.38	14.53	0.37	0.0039	3.24

P13-Lo	44	19.46	4.88	0.10	0.0010	1.50
	89	41.23	8.43	0.18	0.0021	2.73

P14-Lo	48	51.50	11.46	0.25	0.0030	1.18
P14-Up	94	16.19	5.67	0.07	0.0017	2.47

P15-Lo	52	36.01	12.55	0.26	0.0030	2.57
	93	42.14	15.37	0.33	0.0040	2.59
	148	25.29	11.75	0.25	0.0030	3.78
P15-Up	52	18.22	3.35	0.17	0.0058	2.57
	93	20.80	8.00	0.12	0.0020	2.59
	148	21.07	6.25	0.05	0.0016	3.78

P16	145	33.99	11.55	0.28	0.0035	3.47
	184	31.26	10.01	0.23	0.0038	1.81

P17	48	33.88	13.67	0.19	0.0033	3.15
	82	33.30	9.95	0.16	0.0026	2.27

P18	44	15.55	3.98	0.06	0.0007	1.35
	83	18.53	5.05	0.08	0.0009	1.38
